# Systematic Review and Meta-Analysis of Interventions to Improve Access and Coverage of Adolescent Immunizations

**DOI:** 10.1016/j.jadohealth.2016.07.005

**Published:** 2016-10

**Authors:** Jai K. Das, Rehana A. Salam, Ahmed Arshad, Zohra S. Lassi, Zulfiqar A. Bhutta

**Affiliations:** a Division of Women and Child Health, Aga Khan University, Karachi, Pakistan; b Robinson Research Institute, University of Adelaide, Adelaide, Australia; c Centre for Global Child Heath, The Hospital for Sick Children, Toronto, Canada; d Center of Excellence in Women and Child Health, The Aga Khan University, Karachi, Pakistan

**Keywords:** Adolescent health, Immunization, Vaccination, School vaccination, National vaccination, Reminders, National permissive recommendation

## Abstract

Vaccination strategies are among the most successful and cost-effective public health strategies for preventing disease and death. Until recently, most of the existing immunization programs targeted infants and children younger than 5 years which have successfully resulted in reducing global infant and child mortality. Adolescent immunization has been relatively neglected, leaving a quarter of world's population underimmunized and hence vulnerable to a number of preventable diseases. In recent years, a large number of programs have been launched to increase the uptake of different vaccines in adolescents; however, the recommended vaccination coverage among the adolescent population overall remains very low, especially in low- and middle-income countries. Adolescent vaccination has received significantly more attention since the advent of the human papillomavirus (HPV) vaccine in 2006. However, only half of the adolescent girls in the United States received a single dose of HPV vaccine while merely 43% and 33% received two and three doses, respectively. We systematically reviewed literature published up to December 2014 and included 23 studies on the effectiveness of interventions to improve immunization coverage among adolescents. Moderate-quality evidence suggested an overall increase in vaccination coverage by 78% (relative risk: 1.78; 95% confidence interval: 1.41–2.23). Review findings suggest that interventions including implementing vaccination requirement in school, sending reminders, and national permissive recommendation for adolescent vaccination have the potential to improve immunization uptake. Strategies to improve coverage for HPV vaccines resulted in a significant decrease in the prevalence of HPV by 44% and genital warts by 33%; however, the quality of evidence was low. Analysis from single studies with low- or very low–quality evidence suggested significant decrease in varicella deaths, measles incidence, rubella susceptibility, and incidence of pertussis while the impact was nonsignificant for incidence of mumps with their respective vaccines. Further rigorous evidence is needed to evaluate the effectiveness of strategies to improve immunization uptake among adolescents from low- and middle-income countries.

Vaccination programs are among the most successful and cost-effective public health strategies for preventing infections. Until recently, most of the existing immunization programs targeted infants and children younger than 5 years which have successfully resulted in reducing global infant and child mortality [1]. As a result, adolescent immunization has been overshadowed, leaving a quarter of world's population vulnerable to a number of preventable diseases. Estimates suggest that around 35 million American adolescents fail to receive at least one recommended vaccine [2]. In 2012, only half of the adolescent girls in the United States received a single dose of human papillomavirus (HPV) vaccine while merely 43% and 33% received two and three doses, respectively [3]. Missed vaccination opportunities for adolescent vaccination against tetanus, diphtheria, pertussis (TDaP), tetravalent meningococcal conjugate vaccine, and HPV are also common in the United States since adolescents are less likely to utilize preventive care [4].

Infectious and vaccine-preventable diseases disproportionately affect the low- and middle-income countries (LMICs) and disadvantaged populations in high-income countries (HICs). There were an estimated 266,000 deaths from cervical cancer worldwide in 2012, accounting for 7.5% of all female cancer deaths, of which nearly 85% occurred in developing countries [5]. The worldwide prevalence of infection with HPV in women without cervical abnormalities is 11%–12% with higher rates in Sub-Saharan Africa (24%), Eastern Europe (21%), and Latin America (16%) [6]. The proportion of invasive cervical cancer cases is higher in the LMICs with a relatively higher mortality/incidence ratio compared to the HICs [7], [8]. In U.S. settings, African-American girls were less likely to have either initiated or completed the three-dose HPV vaccination series [9]. This warrants an additional focus on adolescents from LMICs and underprivileged populations in HICs as they also deserve a healthy transition into adulthood.

The recommended immunization during adolescence by the World Health Organization includes three doses of hepatitis B (for high-risk groups if not previously immunized), Td booster, one dose of rubella (adolescent girls and/or childbearing-aged women if not previously vaccinated), and two doses of HPV for females (9–14 years) and three doses for those aged 15 years and above [10]. Low immunization rates in adolescents have a wide array of implications: outbreaks of vaccine-preventable diseases, negative effects on quality of life, and increased disease associated costs. Importantly, low immunization rates establish reservoirs of disease in adolescents that can affect others, including high-risk infants, elderly persons, and persons with underlying medical conditions.

Adolescent vaccination is a growing topic that has received significantly more attention since the advent of the HPV vaccine in 2006. In recent years, large number of programs have been launched to increase the uptake of different vaccines in adolescent populations; however, the recommended vaccination coverage among adolescents still remains low. These changes reflect an increased emphasis on the importance of adolescent immunization, but by themselves they will not sufficiently increase awareness or immunization rates [1]. The American Academy of Pediatrics suggests implementing one or more of the strategies including reminder calls, prompts or standing orders, strong provider recommendation, including all recommended vaccination at every visit, provider feedback, educating patients and their parents, addressing costs, and setting up vaccination clinics to increase immunization coverage in adolescents [11].

This article is part of a series of reviews conducted to evaluate the effectiveness of potential interventions for adolescent health and well-being. Detailed framework, methodology, and other potential interventions have been discussed in separate articles [12], [13], [14], [15], [16], [17], [18]. In this article, we systematically reviewed published literature to ascertain the effectiveness of interventions to improve immunization coverage among adolescents.

## Methods

We reviewed all literature published up to December 2014 to identify studies on interventions to improve vaccination coverage. We did not restrict our search to any time limits or geographical settings. For the purpose of this review, the adolescent population was defined as aged 11–19 years; however, since many studies targeted youth along with adolescents, exceptions were made to include studies targeting adolescents and youth. Based on the current recommended vaccines for adolescents [19], search was conducted to identify studies focusing on improving coverage for HPV; measles, mumps, rubella (MMR); TDaP; meningococcal conjugate vaccine; and varicella vaccines among adolescents and youth. Studies were excluded if they targeted age groups other than adolescents and youth or did not report segregated data for the age group of interest. Studies were excluded if the intervention was aimed at comparing the efficacy/effectiveness of different vaccine preparations, assessing changes in antibody titers in individual subjects, or comparing various modes of delivering vaccines without control or baseline data.

Our priority was to select existing randomized controlled trials (RCTs), quasitrials, and before–after studies in which the intervention was directed toward the adolescent and youth and reported immunization coverage outcomes. Search strategy was developed using appropriate keywords, medical subject heading, and free text terms. The following principal sources of electronic reference libraries were searched to access the available data: The Cochrane Library, Medline, PubMed, Popline, LILACS, CINAHL, EMBASE, World Bank's JOLIS search engine, CAB Abstracts, British Library for Development Studies at IDS, the World Health Organization regional databases, Google, and Google Scholar. The titles and abstracts of all studies identified were screened independently by two reviewers for relevance and matched. Any disagreements on selection of studies between these two primary abstractors were resolved by the third reviewer. After retrieval of the full texts of all the studies that met the inclusion/exclusion criteria, data from each study were abstracted independently and in duplicate into a standardized form. Studies that met the inclusion criteria were selected and double data abstracted on a standardized abstraction sheet. Quality assessment of the included RCTs was done according to the Cochrane risk of bias assessment tool. We conducted a meta-analysis for individual studies using the software Review Manager, version 5.3 (Cochrane Collaboration, London, United Kingdom). Pooled statistics were reported as the relative risk (RR) for categorical variables and standard mean difference for continuous variables between the experimental and control groups with 95% confidence intervals (CIs). A grade of “high,” “moderate,” “low,” and “very low” was used for grading the overall evidence indicating the strength of an effect on specific health outcome according to the Grading of Recommendations Assessment, Development and Evaluation criteria [20].

## Results

**Figure 1:**
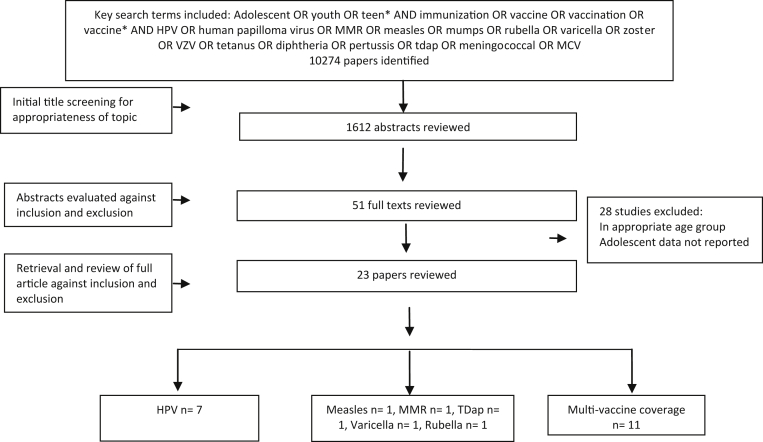
Search flow diagram. MCV = meningococcal conjugate vaccine; VZV = varicella zoster vaccine.

Figure 1 describes the search flow while characteristics of the included studies are detailed in Table 1. The search yielded 10,274 titles across all databases that were screened for the purpose of this review. Screening the relevant abstracts resulted in 51 full texts that were further screened after which 23 studies were included in this review [21], [22], [23], [24], [25], [26], [27], [28], [29], [30], [31], [32], [33], [34], [35], [36], [37], [38], [39], [40], [41], [42], [43], of which four were RCTs, three quasirandomized trials, and 16 before–after studies. Of the 23 included studies, seven [26], [27], [28], [29], [31], [32] focused on the HPV, 11 studies [33], [34], [35], [36], [37], [38], [39], [40], [41], [42], [43] implemented interventions to improve coverage of multiple vaccines recommended for adolescents while measles [21], MMR [22], varicella [23], rubella [25], and TDaP [24] vaccines were assessed in one study each. All the studies were conducted in HICs of the United States, Canada, Australia, Denmark, and England. Included studies mainly focused on evaluating the impact of licensing and mass provision of vaccines as a part of national-level vaccination program to increase provision and coverage of adolescent vaccination, availability of free vaccines, implementing vaccination requirement before entry into school, reminder letters, telephone calls, and training of clinic staff on adolescent vaccines and strategies to improve immunization rates. Target populations in all studies were adolescents aged 11–19 years except three studies in the HPV vaccine category that targeted adolescents and youth till the age of 24 years.

**Figure 2:**
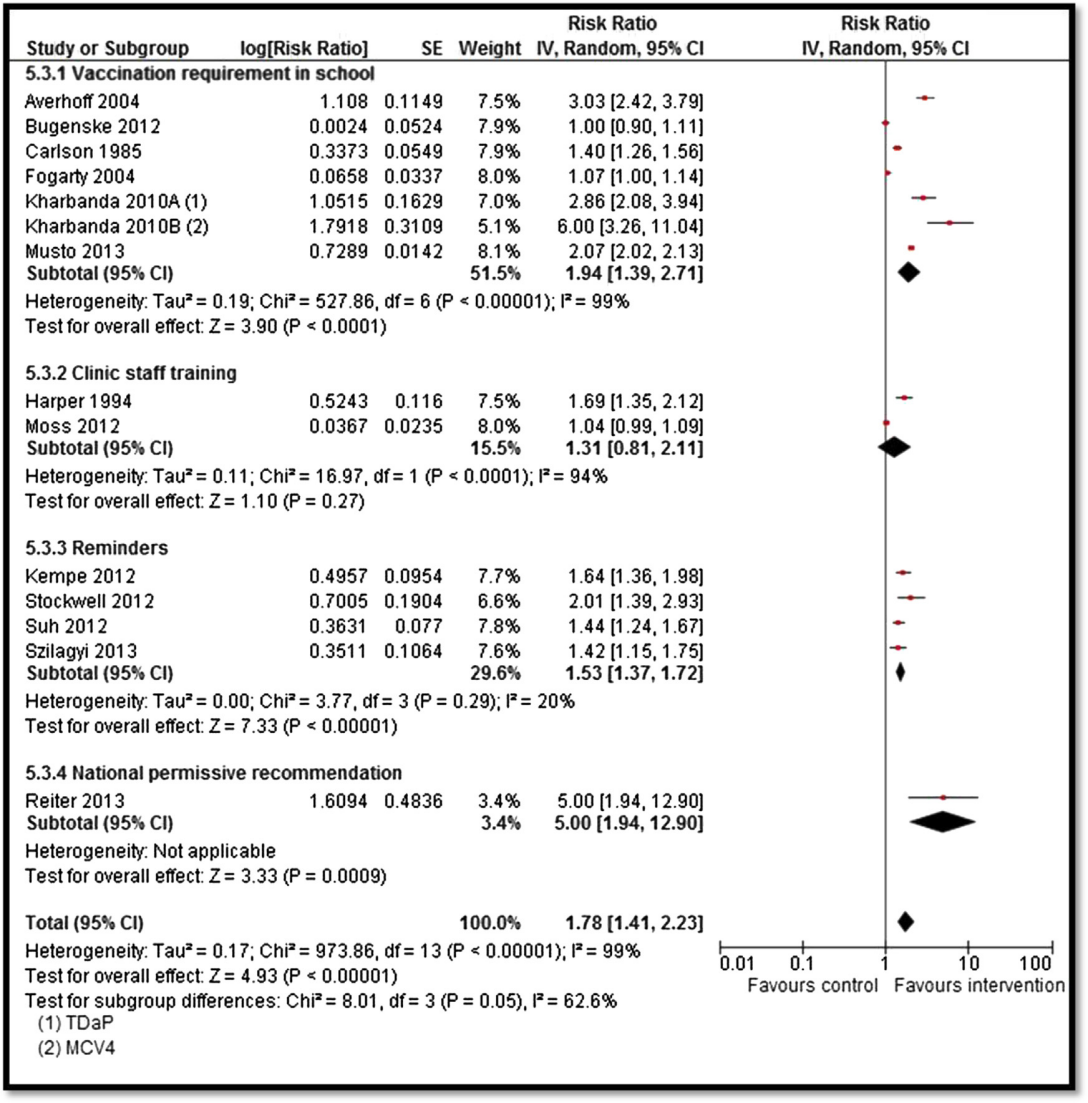
Forest plot for the impact of strategies on vaccination coverage. IV = inverse variance; MCV = meningococcal conjugate vaccine; SE = standard error.

**Figure 3:**
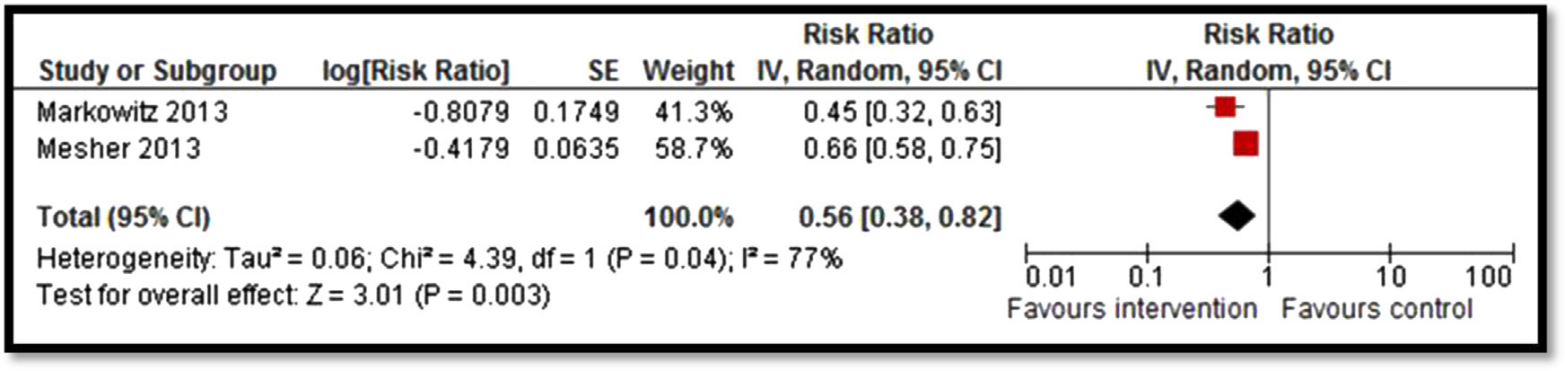
Forest plot for the prevalence of HPV. IV = inverse variance; SE = standard error.

**Figure 4:**
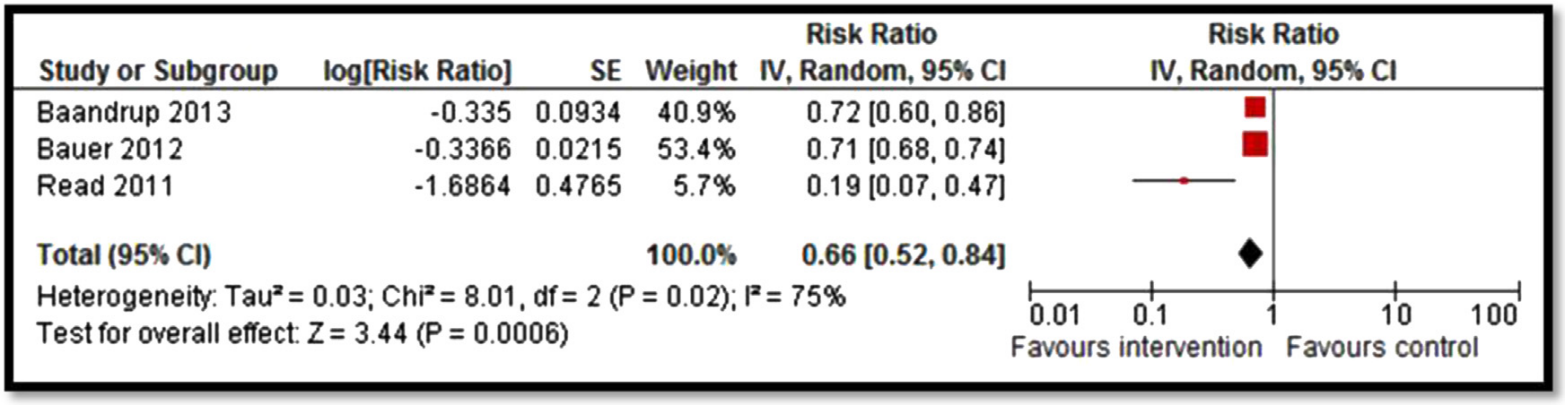
Forest plot for the prevalence of genital warts. IV = inverse variance; SE = standard error.

Moderate-quality evidence from 13 studies suggested an overall increase in vaccination coverage by 78% (RR: 1.78; 95% CI: 1.41–2.23; Figure 2) [30], [33], [34], [35], [36], [37], [38], [39], [40], [41], [42], [43]. Subgroup analysis suggests that vaccination requirement in school, reminders, and national permissive recommendation had a significant impact on improving coverage while clinic staff training showed a nonsignificant impact. Strategies to improve coverage for HPV vaccines including countrywide provision and clinic-based delivery resulted in a significant decrease in the prevalence of HPV by 44% (RR: .56; 95% CI: .38–.82; Figure 3) [28], [29] and genital warts by 33% (RR: .66; 95% CI: .52–.84; Figure 4) [26], [27], [31]; however, the quality of evidence was low. Since only one study each was included for measles, mumps, pertussis, and varicella vaccines, it was not possible to pool results. Analysis from single studies with low or very low quality suggested significant decrease in varicella deaths (RR: .74; 95% CI: .56–.98), measles incidence (RR: .12; 95% CI: .03–.38), rubella susceptibility (RR: .27; 95% CI: .15–.46), and incidence of pertussis (RR: .24; 95% CI: .16–.36) while the impact was nonsignificant for incidence of mumps (RR: .96; 95% CI: .42–2.18).

The outcome quality was rated to be “moderate” for vaccine coverage; “low” for the prevalence of HPV, genital warts, and mump incidence; and “very low” for varicella deaths, measles incidence, rubella susceptibility, and incidence of pertussis. The outcome quality was downgraded due to nonrobust designs, heterogeneity, and limited generalizability to HICs only. A summary of quality of evidence is provided in Table 2.

## Discussion

Our review findings suggest that strategies to increase HPV, TDaP, MMR, and varicella vaccination uptake among adolescents can significantly improve the coverage for these vaccines. Implementing vaccination requirement in school, sending reminders, and national permissive recommendation for adolescent vaccination has the potential to improve immunization uptake. These interventions have also led to significant decline in the prevalence of HPV and genital warts; incidence of measles and pertussis; rubella susceptibility; and varicella deaths. However, these findings should be interpreted with caution since these are from single studies with low or very low quality. Furthermore, these studies capture the incremental benefits of vaccination of those who may have missed earlier doses or failed to seroconvert to earlier doses since these vaccines are usually given at younger ages.

All the included studies were conducted in HICs depicting dearth of evidence evaluating the effectiveness of strategies to improve immunization uptake among adolescent from LMICs. This could also be attributable to the scope of review since our review was restricted to strict inclusion and exclusion criteria, and we did not include gray literature reporting various country case studies. Furthermore, recent state mandatory vaccination and exception policies could also have affected the vaccination coverage rates; however, these programs and policy interventions do not lend themselves to intervention studies. One of the limitations of the review was that the search terms were in English, and hence foreign language articles may not have been identified. There is lack of rigorously designed studies since most of the existing studies have utilized the pre- and postimplementation data after the approval of vaccine legislation or national launch of vaccination program without having a control site. Only a single study each for MMR, TDaP, varicella, and meningococcal vaccines were found, showing a lack of focus evaluating the impact of uptake for vaccines other than HPV. This highlights the need for further studies to assess the uptake and delivery platforms to deliver these vaccines in adolescent population. Included studies targeted various overlapping adolescent and youth age groups that might have led to variations in the outcome effect.

Despite the high burden of infectious diseases and low immunization coverage in LMICs, strategies to improve vaccine coverage for adolescent age group are minimal. Although there are existing data outlining what exists in LMICs for delivering adolescent immunization, primarily through school-based approaches; however, there are little data that have systematically been evaluated for the impact of strategies to increase coverage [44]. Various countries' case studies have documented experiences from LMICs with existing school-based immunization programs, for example, in Indonesia, Malaysia, Sri Lanka, and Tunisia; however, they lack rigorous evaluations [45]. For HPV, various national-level programs are in place especially in LMICs; Bhutan is the first LMIC to roll out a national HPV vaccination program, followed by Rwanda and Uganda. These programs suggest that vaccine uptake can be improved by providing evidence-based education and outreach; however, experiences in these countries underscore complex challenges and planning to ensure sustainability [46]. The number of LMICs that have introduced HPV vaccination is relatively low; however, the coverage levels in these countries are relatively higher than in some HIC. Enabling factors for improved coverage in LMICs include political will, nationwide sensitization campaign, school-based vaccination, and community involvement [44], [45], [46], [47].

Despite the availability of the HPV vaccine in HICs like the United States, the uptake remains low. Vaccine utilization is a multifactorial phenomenon which depends on several factors including vaccine acceptability, perceived disease susceptibility, perceived benefit of vaccination, and intention to receive the particular vaccine. A recent systematic review on barriers to HPV vaccination among adolescents in the United States suggests financial concerns and parental attitudes as barriers to HPV vaccination [48], [49]. Good understanding and knowledge of the factors and importance of vaccine in target population are important for tailoring vaccine improvement strategies and subsequent success of the program in achieving targeted vaccine coverage [50]. It is imperative to develop and test context-specific strategies to improve adolescent vaccine uptake and dose completion rates. Educational interventions could increase knowledge and clear misconceptions related to seriousness of vaccine-preventable infection and cervical cancer, susceptibility of adolescents to infection, and risk of infection. Such strategies would also address barriers to adolescent vaccine uptake and dose completion, such as parental concerns about vaccine safety, and effectiveness [51]. Very few of the included studies in our review utilized mHealth/eHealth technology for improving immunization coverage which could be one of the potentially effective strategies to target adolescent age group especially in LMIC settings owing to the higher use in this age group and recent explosion in Internet access in developing countries due to the emergence of mobile Internet [52]. One of the concerns with the introduction of HPV vaccine in low-resourced high-burden countries is lack of cost-effective data; however, some recent analysis suggests that HPV vaccination is likely to be cost-effective, especially in LMICs [53], [54].

Improving vaccination coverage to decrease the burden of these preventable diseases would require an integrated approach ranging from mass availability of vaccines at the national level to targeting adolescents in school and during health care visits to optimize the effectiveness of immunization programs. Besides these programs, there is a need for an increased emphasis on the importance of adolescent immunization by identifying and overcoming barriers to adolescent vaccination. Further research is needed to explore why missed vaccination opportunities occur and to develop evidence-based strategies to reduce missed opportunities and improve adolescent vaccination coverage.

## Figures and Tables

**Table 1:** Characteristics of included studies

Study	Study design	Country	Setting	Intervention	Target population	Control	Outcomes assessed
Measles							
Zhuo et al. [21]	Before–after	China	Community	Supplementary immunization activities	All ages	No supplementary immunization activity	Incidence of measles
MMR							
Ogbuanu et al. [22]	Before–after	United States	School	Selective school-based immunization	9–14 years	No intervention	Incidence of mumps
Varicella							
Nguyen et al. [23]	Before–after	United States	Nationwide vaccination	Universal childhood varicella vaccination program	All ages (outcome assessed for 10–19 years)	Before implementation of childhood varicella immunization	Number of deaths caused by varicella infection
TDaP							
Quinn and McIntyre [24]	Before–after	Australia	School	School-based delivery of TDaP	12–19 years	Nonavailability of school-based immunization	Incidence of pertussis
Rubella							
Nelson et al. [25]	Before–after	United States	School	Vaccination requirement in school	Girls older than 10 years	Before the vaccination requirement	Rubella susceptibility
HPV							
Baandrup et al. [26]	Before–after	Denmark	Countrywide provision	Licensing and mass provision of HPV as part of National HPV Program	12–19 years	Absence of nationwide HPV availability	Incidence of genital warts
Bauer et al. [27]	Before–after	United States	Countrywide provision	Introduction population-level administration of HPV vaccine	<21 years	Nonavailability of population-level vaccination	Incidence of genital warts
Markowitz et al. [28]	Before–after	United States	Countrywide provision	Introduction of HPV vaccine into routine immunization schedule	14- to 24-year-old females	HPV vaccine not included in routine immunization schedule	Prevalence of HPV
Mesher et al. [29]	Before–after	England	Countrywide provision	Introduction of National HPV Immunization Program	16- to 24-year-old females	Nonavailability of population-level vaccination	Prevalence of HPV
Musto et al. [30]	Quasitrial	Canada	School and community	Within schools vaccination during Grades 1, 5, and 9	9- to 11- and 13- to 15-year-old females	Community-based vaccine availability at local community clinics by appointment	Vaccine uptake
Read et al. [31]	Before–after	Australia	Clinic	Introduction of National HPV Vaccination Program	12- to 18-year-old females	HPV vaccine not included in national immunization schedule	Incidence of genital warts
Reiter et al. [32]	Before–after	United States	Nationwide recommendation	National permissive recommendation for HPV vaccine	11- to 17-year-old males	No recommendation	Vaccine initiation
Multivaccine							
Averhoff et al. [33]	Before–after	United States	School	Vaccination requirement in school	Fifth- through eighth-grade students	Students not subject to the requirement	Vaccine coverage
Bugenske et al. [34]	Quasitrial	United States	School	Vaccination requirement in school	13–17 years	Students not subject to the requirement	Vaccine coverage
Carlson and Lewis [35]	Before–after	Canada	School	Vaccination requirement in school	Grades 7–13	Students not subject to the requirement	Vaccine coverage
Fogarty et al. [36]	Before–after	United States	School	Vaccination requirement in school	Seventh-grade students	No control	Vaccine coverage
Harper and Murray [37]	Quasitrial	United States	Clinic	Clinic staff recommended vaccine on every visit	11–18 years	No recommendation	Vaccine coverage
Kempe et al. [38]	RCT	United States	School	Recall reminders for vaccination	Sixth-grade male students	No recommendation	Vaccine coverage
Kharbanda et al. [39]	Before–after	United States	Hospital	Vaccination requirement in school	11–14 years	Students not subject to the requirement	Vaccine coverage
Moss et al. [40]	Before–after	United States	Clinic	Clinic staff were invited to attend 1-hour one-on-one webinar on adolescent vaccines and strategies to improve immunization rates such as reviewing and flagging charts, decreasing missed opportunities, recalls, and establishing center guidelines for immunizations.	12–17 years	No staff training	Vaccine coverage
Stockwell et al. [41]	RCT	United States	Community	Text message reminders for vaccination	12–18 years	No reminder	Vaccine coverage
Suh et al. [42]	RCT	United States	Clinic	Reminders letters and calls for vaccination	11–18 years	No reminder	Vaccine coverage
Szilagyi et al. [43]	RCT	United States	Clinic	Mail letters and telephone reminders for vaccination	11–17 years	No reminder	Vaccine coverage

HPV = human papillomavirus; MMR = measles, mumps, rubella; RCT = randomized controlled trial; TDaP = tetanus, diphtheria, pertussis.

**Table 2:** Summary of findings for the effect of interventions for improving immunization coverage among adolescents

Quality assessment						Summary of findings		
Number of studies	Design	Limitations	Consistency	Directness		Number of events		RR (95% CI)
				Generalizability to population of interest	Generalizability to intervention of interest	Intervention	Control	
Vaccine coverage: moderate outcome-specific quality of evidence								
13 studies (14 data sets)	RCT, quasi, and observational studies	Study designs not robust	Twelve studies suggest benefit	All studies targeted adolescents aged 11–19 years in developed countries	Interventions included vaccination requirement in school, reminders, and national permissive recommendation	5,092	4,303	1.78 (1.41–2.23)
HPV prevalence: low outcome-specific quality of evidence								
Two studies	Observational studies	Study designs not robust	Both studies suggest benefit	Studies targeted adolescents aged 14–24 years in developed countries	Intervention included introducing HPV vaccine into routine immunization	499	554	.56 (.38–.82)
Incidence of genital warts: low outcome-specific quality of evidence								
Three studies	Observational studies	Study designs not robust	All three studies suggest benefit	All studies from developed countries targeting adolescents from age 12 to 21 years	All studies focused on increased provision of HPV vaccine through national HPV programs	3,875	5,409	.66 (.52–.84)
Varicella deaths: very low outcome-specific quality of evidence								
One	Observational study	Study design not robust	Only one study	Intervention targeted all age groups in the United States, outcomes reported for 10- to 19-year age group	Universal childhood varicella vaccination program	77	104	.74 (.56–.98)
Mumps incidence: low outcome-specific quality of evidence								
One	Quasitrial	No randomization (quasitrial)	Only one study	Adolescents 9–14 years in the United States	School-based immunization	28	7	.96 (.42–2.21)
Pertussis incidence: very low outcome-specific quality of evidence								
One	Observational study	Study design not robust	Only one study	Interventions targeted adolescents 12–19 years in Australia	School-based delivery of TDaP vaccine	31	128	.24 (.16–.36)
Rubella susceptibility: very low outcome specific quality of evidence								
One	Observational study	Study design not robust	Only one study	Interventions targeted adolescent girls >10 years in the United States	Vaccination requirement in school	15	49	.27 (.15–.46)
Measles incidence: very low outcome-specific quality of evidence								
One	Observational study	Study design not robust	Only one study	Interventions targeted all ages in China	Supplementary immunization activities	3	26	.12 (.03–.38)

CI = confidence interval; HPV = human papillomavirus; RCT = randomized controlled trial; RR = relative risk; TDaP = Tetanus, diphtheria, pertussis.
